# Red nucleus structure and function: from anatomy to clinical neurosciences

**DOI:** 10.1007/s00429-020-02171-x

**Published:** 2020-11-12

**Authors:** Gianpaolo Antonio Basile, Marina Quartu, Salvatore Bertino, Maria Pina Serra, Marianna Boi, Alessia Bramanti, Giuseppe Pio Anastasi, Demetrio Milardi, Alberto Cacciola

**Affiliations:** 1 Brain Mapping Lab, Department of Biomedical, Dental Sciences and Morphological and Functional Images, University of Messina, Messina, Italy; 2 Section of Cytomorphology, Department of Biomedical Sciences, University of Cagliari, Cittadella Universitaria di Monserrato, 09042 Monserrato, CA Italy; 3 IRCCS Centro Neurolesi “Bonino Pulejo”, Messina, Italy

**Keywords:** Locomotion, Neuroimaging, Pain, Phylogenesis, Review, Skilled movements

## Abstract

The red nucleus (RN) is a large subcortical structure located in the ventral midbrain. Although it originated as a primitive relay between the cerebellum and the spinal cord, during its phylogenesis the RN shows a progressive segregation between a magnocellular part, involved in the rubrospinal system, and a parvocellular part, involved in the olivocerebellar system. Despite exhibiting distinct evolutionary trajectories, these two regions are strictly tied together and play a prominent role in motor and non-motor behavior in different animal species. However, little is known about their function in the human brain. This lack of knowledge may have been conditioned both by the notable differences between human and non-human RN and by inherent difficulties in studying this structure directly in the human brain, leading to a general decrease of interest in the last decades. In the present review, we identify the crucial issues in the current knowledge and summarize the results of several decades of research about the RN, ranging from animal models to human diseases. Connecting the dots between morphology, experimental physiology and neuroimaging, we try to draw a comprehensive overview on RN functional anatomy and bridge the gap between basic and translational research.

## Introduction

The human red nucleus (RN) is a large subcortical structure located in the ventral midbrain, which is cytoarchitectonically divided into two histologically distinct subregions: a magnocellular, caudal region, consisting of large sparse neurons (magnocellular RN, mRN) and a rostral parvocellular part, mainly characterized by small and medium-sized neurons (parvocellular RN, pRN) (Ulfig and Chan 2002; Yamaguchi and Goto 2008; Onodera and Hicks 2009, 2010; Paxinos et al. 2012). These structures play a complementary role in different aspects of motor control (Kennedy 1990), and are likely involved in different motor disorders, such as essential tremor (ET) (Wills et al. 1994, 1995), Parkinson’s disease (PD) (Wang et al. 2016b; Guan et al. 2017) and in the recovery from pyramidal lesions (Yeo and Jang 2010; Rüber et al. 2012; Takenobu et al. 2014; Jang and Kwon 2015; Kim et al. 2018). Despite its remarkable clinical interest, the RN still remains a poorly investigated region of the human brain.

We have therefore explored different grounds of research that, ranging from phylogenesis, comparative anatomy (ten Donkelaar 1988; Onodera and Hicks 2009), experimental physiology (Kennedy et al. 1986; Kennedy and Humphrey 1987; Mewes and Cheney 1994; Belhaj-Saïf et al. 1998), to recent brain imaging studies, both in healthy subjects and in neurological disorders (Wills et al. 1994; Nioche et al. 2009; Rüber et al. 2012; Lewis et al. 2013; Zhang et al. 2015a; Milardi et al. 2016; de Hollander et al. 2017; Belkhiria et al. 2018; Cacciola et al. 2019), may represent the basis to reach a better understanding of this integrative region of the brain.

The mRN and pRN are segregated not only cytoarchitectonically, but also on the basis of their connections. The mRN, that is the phylogenetically older region, contains efferent neurons whose axons, after crossing the midline, project mainly to the spinal cord, whereas the pRN sends its major projection to the inferior olive (Papez and Stotler 1940; Nathan and Smith 1982; Onodera and Hicks 2009, 2010). These distinct regions of RN show high morphological and functional variability across different species (ten Donkelaar 1988; Onodera and Hicks 2009) and, currently, most of our present knowledge comes from experiments carried out in animal species as different as rodents and primates (Gruber and Gould 2010), though the RN of these animal models shows striking diversities from one another and, crucially, from the human RN: this represents an important limitation in translating animal findings to human research.

The most paradigmatic example of such differences is the regression of the mRN, that is the best characterized region of RN in quadrupedal animals, whilst it is generally considered a kind of a vestige with unknown functional relevance in humans (Nathan and Smith 1982; Patt et al. 1994). By contrast the pRN, whose functional role remains largely unknown in most experimental models (Kennedy et al. 1986; Kennedy and Humphrey 1987), is much more delineated in non-human and human primates, suggesting that it could have gained considerable importance in some key features of brain development in humans, and that only human research could provide us a complete view about its functions. However, available data on human RN have been, to date, mostly fragmentary.

The last 30 years have been characterized by the rise of neuroimaging techniques, such as structural MRI, functional MRI (fMRI), diffusion tensor imaging and tractography, which have rapidly affirmed as powerful tools for studying the human brain in vivo and non-invasively. Different neuroimaging studies examining the human RN in both physiological and pathological conditions, suggest that, in humans, it could be involved not only in motor control but also in sensory processing and higher-order cognitive functions (Habas et al. 2010). However, these results are not always consistent and make often difficult to allow a clear-cut interpretation.

Hence, as we succinctly outlined, many open questions still remain about RN: if on one hand it is obvious that the human RN shows relevant structural differences when compared to the RN of the most studied animal models, thus preventing direct translation of findings derived from these models to the human brain, on the other hand the claims for qualitative functional differences between humans and all the other species are supported by limited evidence.

Aimed at providing a comprehensive overview on RN functional and clinical anatomy, the present work reviews the existing literature, tracing a line between morphology, experimental physiology, functional and structural neuroimaging, discussing past and current models of RN anatomy and physiology, underlining critical issues and suggesting possible future directions of research. We attempt to define a unified conceptual framework in which structural and functional properties of the human RN, as emerging from anatomy, physiology and neuroimaging studies, are interpreted in light of the existing knowledge on the evolution of RN among different animal species. In addition, pathophysiological and clinical implications are examined in order to bridge the gap between basic and translational research. Finally, we emphasize the need for further research to improve our current knowledge on this neglected brain structure.

## RN across evolution: a phylogenetic perspective

Analyzing RN morphology within a comparative, phylogenetic framework is the first step to understand how and why this structure is so different between animal species and humans.

A classical review by ten Donkelaar (1988) summarizes the earliest steps of this evolutionary process. The core concept behind the paper is that, in most of vertebrate phyla, major changes in RN cytoarchitecture and connectivity coincide with major changes in motor behavior, and in particular in locomotion patterns. In line with this postulate, a primitive RN is almost invariably present in animals having fins, wings, limbs or limb-like structures as a mean of locomotion, while it is absent in primitive vertebrates, boid snakes and sharks (ten Donkelaar 1976; Ten Donkelaar^1976^; Smeets and Timerick 1981; Ten Donkelaar and Bangma 1983; ten Donkelaar et al. 1983). It appears in rays, which use their large pectoral fins for locomotion, and is maintained in limbed amphibians, such as anurans and frogs (Ten Donkelaar et al. 1980; Corvaja and D’Ascanio 1981). Notably, in the tadpoles of the anuran *Xenopus levis*, the ontogenesis of the connections between the primitive RN and the spinal cord coincides with the transition from the aquatic stage to amphibian stage, and the development of structured limbs (ten Donkelaar and de Boer-van Huizen 1982).

**Fig. 1:**
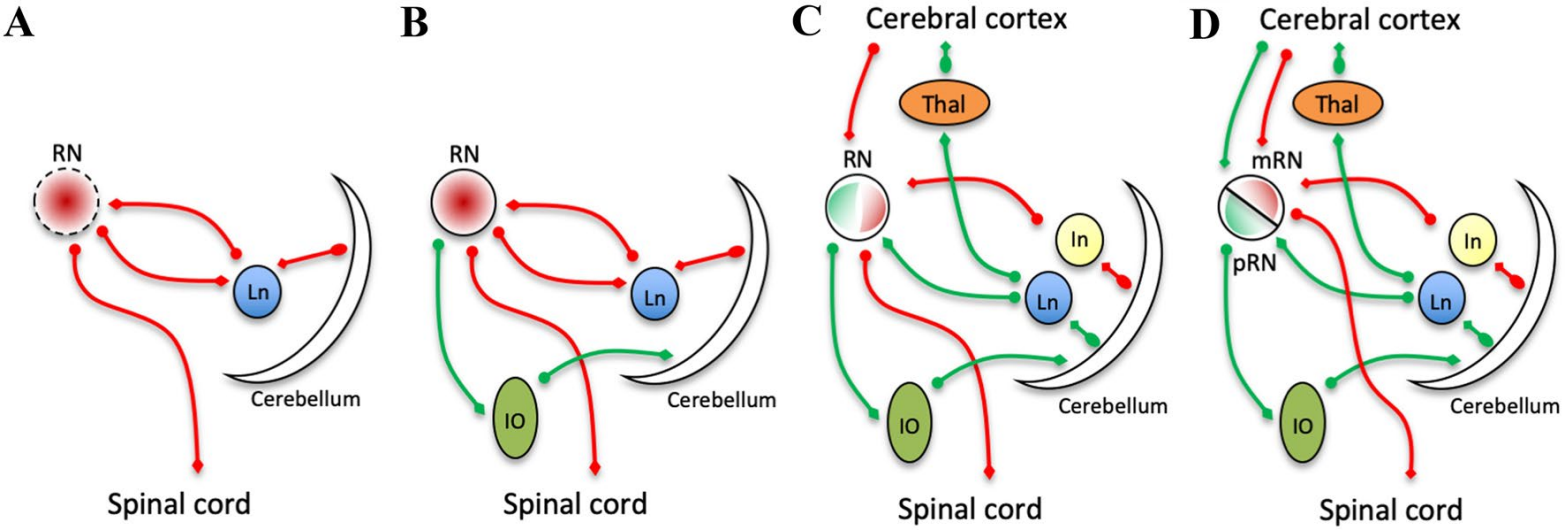
Evolution of the RN circuitry. The scheme highlights the increasing complexity of rubral circuitry, and the evolutionary trend towards gradual segregation of the rubrospinal (red) and rubro-olivo-cerebellar (green) systems. **a** Primitive RN (anurans) is a small, ill-delimited group of neurons in the ventral midbrain (dashed borders), representing a rather simple relay station between the cerebellum and the spinal cord. **b** In quadrupedal reptiles, a rubro-olivary pathway appears. **c** In quadrupedal mammals, a partial segregation between rubrospinal and rubro-olivo-cerebellar systems occurs, with distinct cerebellar output channels and a gradual differentiation between parvocellular and magnocellular RN. **d** In primates, complete anatomical segregation and functional specialization of rubrospinal and rubro-olivo-cerebellar systems can be observed. *RN* red nucleus, *mRN* magnocellular red nucleus, *pRN* parvocellular red nucleus, *Ln* lateral nucleus (dentate), *In* interposed nucleus, *IO* inferior olive, *Thal* thalamus

From an evolutionary point of view, the RN is likely to be involved in the transition from the swimming pattern of aqual cordates to the land locomotion of terrestrial vertebrates (ten Donkelaar 1988; Gruber and Gould 2010). This evolutionary drive may explain the trend of increasing complexity and progressive structural segregation and specialization, observed across different species (Fig. 1). In primitive vertebrates and amphibians, the RN consists of just a few cells that receive efferents from the cerebellum and reach the contralateral spinal cord through the rubrospinal tract (RST) (Corvaja and D’Ascanio 1981; ten Donkelaar and de Boer-van Huizen 1982; Gonzalez et al. 1984) (Fig. 1a). In four-limbed reptiles, such as the lizard *Varanus exanthematicus*, a small and rudimentary ipsilateral rubro-olivary projection appears (ten Donkelaar and de Boer-van Huizen 1981), together with an olivary projection to the contralateral cerebellum (ten Donkelaar and de Boer-van Huizen 1982). Such an advanced level of organization likely reflects the need for a wider repertory of skilled limb movements in comparison to simple, repetitive behaviors that are typical of lower vertebrates (ten Donkelaar 1988) (Fig. 1b).

In mammals, the development of the cerebellum and cerebral cortex, along with the corticospinal tract taking the place of the reticulospinal tract in controlling spinal motor neurons, is thought to reflect the evolutionary need for more complex locomotion patterns (Shapovalov 1972, 1975). In line with this theory, in the American opossum *Didelphis virginiana,* a primitive marsupial mammal that uses its upper limbs to climb on trees, RN anatomy shows a third level of organization: two distinct regions (approximately a caudal and a rostral portion) are defined by afferent input from distinct deep cerebellar nuclei (anterior interpositus and lateral nucleus respectively, the latter being the homologue of the human dentate) (Martin et al. 1974). Another sign of regional specialization is represented by projections from the ipsilateral motor cortex, targeting mostly (but not exclusively) the rostral two thirds of the RN (King et al. 1972; Martin et al. 1974, 1983).

This gradual tendency towards diversification becomes more evident in the RN of rodents, which is one of the best characterized morphofunctional models. Rodent’s RN is a cytoarchitectonic continuum of different sized and shaped cells, and although there are not clearly distinguishable internal boundaries between the magnocellular and parvocellular portions, a well recognizable gradient of distribution can still be identified: small and medium-sized cells are more represented in the rostral part of RN, while giant and large cells in the caudal part. According to their morphology, three cell types can be identified: (i) intrinsic achromatic Golgi-type II interneurons; (ii) “magnocellular” neurons with coarse Nissl body pattern; (iii) “parvocellular” neurons with fine-grained Nissl body pattern (Reid et al. 1975; Liang et al. 2012). Thus, in rodents, differences between cell types reflect differences in terms of efferent connectivity: magnocellular “coarse” neurons project to the contralateral spinal cord reaching the lumbar enlargement (Strominger et al. 1987), while small “fine-grained” cells project to the inferior olivary nucleus (Swenson and Castro 1983a, b; Kennedy and Humphrey 1987). Similar differences are evident also for afferent connectivity patterns: lateral cerebellar nucleus (dentate) projects mainly to the rostral RN, whilst anterior and posterior interposed nuclei to the caudal RN (Angaut et al. 1986; Daniel et al. 1988; Ruigrok 2004), delimiting some kind of partially segregated “magnocellular” and “parvocellular” territories. Noteworthy, this functional segregation is not complete, as some “fine-grained” parvocellular cells also contribute to the rubrospinal tract (Huisman et al. 1981, 1983; Shieh et al. 1983; Kennedy 1990); on the other hand, direct projections from cerebral cortex involve exclusively the rostral parvocellular part (Brown 1974; Gwyn and Flumerfelt 1974). There is, in addition, evidence of a somatotopical organization of rubral neurons in relation to their projections to the spinal cord: indeed, medial neurons project to cervical spinal cord, whereas ventrolateral ones to the lumbar cord (Murray and Gurule 1979; Shieh et al. 1983). Such a topographical organization is maintained also in projections from the anterior interposed nucleus, that shows a ventro-dorsal somatotopy (Daniel et al. 1988).

In cats, the organization of the RN is very similar to that described in the rat and in the opossum: the parvocellular region is mostly connected to the inferior olive and the dentate nucleus, whereas the magnocellular region to the anterior interposed nucleus and spinal cord (Pompeiano and Brodal 1957; Courville 1966; Holstege and Kuypers 1987; Holstege and Tan 1988; De Zeeuw et al. 1990; Onodera and Hicks 2009); connectivity differences between these regions are even less marked and, as for the opossum, direct cortico-rubral projections from motor cortex also involve the magnocellular caudal pole of RN (Giuffrida et al. 1988). In addition, a large extent of parvicellular neurons share the same connectivity features of magnocellular neurons (to interposed nucleus and spinal cord) and evidence of a proximal-vs-distal organization has been found for the spinal output of parvocellular and magnocellular neurons, respectively (Pong et al. 2002). Finally, a direct ascending spino-rubral projection conveying somatosensory information to the RN has been also described (Padel et al. 1986, 1988) (Fig. 1c).

In primates, the structural segregation between the magnocellular and parvocellular parts of RN is patent, as these structures show distinct regional distribution and connectional anatomy: magnocellular neurons with coarse Nissl pattern occupy exclusively the caudal portion of the RN, while parvocellular (fine-grained) neurons are confined to the rostral and ventral portions, forming two easily recognizable substructures: the mRN and pRN (Massion 1967, 1988; Miller and Strominger 1973; Onodera and Hicks 2009). The marked differences of these substructures become more evident also in their structural connectivity profiles: mRN receives its main afferent projection from the interposed nucleus (Asanuma et al. 1983; Kennedy et al. 1986) and gives rise to the crossed rubrospinal tract, which reaches the spinal cord (Castiglioni et al. 1978). Conversely, the pRN receives afferent fibers from the dentate nucleus (Flumerfelt et al. 1973; Stanton 1980) and its exclusive efferent projection is conveyed to the ipsilateral inferior olive (Miller and Strominger 1973; Robertson and Stotler 1974). The vast majority (approximately 90%) of direct projections from bilateral motor, premotor and supplementary motor cortices are directed to the pRN, with only a smaller contingent from ipsilateral motor cortex reaching the mRN (Kuypers and Lawrence 1967; Humphrey and Rietz 1976; Humphrey et al. 1984; Ralston 1994; Tokuno et al. 1995; Onodera and Hicks 2009; Lemon 2016) (Fig. 1d). The projections from primary motor cortex are somatotopically organized with a medio-lateral gradient (Murray and Haines 1975; Larsen and Yumiya 1980) and a similar topographical organization is also described for projections from the supplementary motor area (SMA) (Tokuno et al. 1995).

Some preliminary functional considerations can be drawn from this phylogenetic model.

First, the evolution of RN moved toward the gradual segregation and specialization of two distinct neural circuits: a rubrospinal system (mRN and related circuitry) and a rubro-olivo-cerebellar system (pRN and related circuitry). The former is the expression of a phylogenetically older motor control system, depending more from the cerebellum than from the cerebral cortex. The latter appeared for the first time in quadrupedal lizards, and reached its most complete development in quadrupedal mammals, where the development of cerebral cortex and its increasing importance in motor control led to the appearance of cerebello-cortical and cortico-rubral projections. In primates also, the gradual transition from quadrupedal to bipedal stance marked the gradual regression of mRN and the progressive enlargement of pRN. A well-developed mRN can be identified in monkeys, such as macaques and baboons, which use quadrupedal gait during locomotion (Miller and Strominger 1973; Padel et al. 1981). By contrast, increasingly bipedal apes, gibbons and chimpanzees show a smaller mRN (Padel et al. 1981; Massion 1988). Hence, it is likely that such a structural regression of mRN may subserve a further functional specialization, in relation to a new evolutionary drive: the need for complex upper limb motility and the development of hands.

## From locomotion to hand skilled movements: the evolution of the rubrospinal system

As seen before, the rubrospinal system is the phylogenetically older functional unit of the RN. In earlier vertebrates it consists of a simple circuit in which magnocellular neurons act as a relay between the cerebellar interposed nucleus and the contralateral spinal cord through the RST (Corvaja and D’Ascanio 1981; Gonzalez et al. 1984).

Magnocellular neurons can be considered as primitive motoneurons: if stimulated singularly (microstimulation), each of them is able to elicit contraction of single muscular units (Ghez 1975).

In rats (Jarratt and Hyland 1999), cats (Ghez and Kubota 1977; Burton and Onoda 1978; Padel and Steinberg 1978; Amalric et al. 1983; Batson and Amassian 1986) and primates (Gibson et al. 1985a; Kennedy et al. 1986; Mewes and Cheney 1991, 1994; Miller and Houk 1995), activity of rubrospinal neurons show a striking correlation with the execution of voluntary movements by forelimbs and hindlimbs, as burst firing activity usually precedes or follows the onset of movements. Experiments in a turtle in vitro model of mRN activation support the hypothesis of a positive feedback between RN and interposed nucleus, as the selective inactivation of RN reduces activity in interposed nucleus and vice-versa (Keifer 1996); this would suggest that burst discharge initiation depends on activation of the interposed nucleus (Toyama et al. 1970). In primates, neuronal activity of rubrospinal neurons strongly correlates with timing and magnitude of upper limb muscular activity (Miller et al. 1993; Mewes and Cheney 1994; Miller and Houk 1995) and encodes both kinematic (velocity-related) and dynamic (force-related) parameters of upper limb movements (Kohlerman et al. 1982; Kennedy 1987; Cheney et al. 1988).

Interestingly, lesions of the rubrospinal tract lead to a more marked motor impairment in distal rather than in proximal limb muscles (Lawrence and Kuypers 1968). Indeed, in both cats and primates, burst activation of mRN is stronger in movements involving distal rather than proximal limbs (Ghez and Kubota 1977; Burton and Onoda 1978; Ghez and Vicario 1978; Amalric et al. 1983; Mewes and Cheney 1991). In cats, where a consistent part of the RST is formed by cells from pRN, a proximal-vs-distal topographical organization of spinal outputs has been found: mRN acts mostly on distal muscles, while pRN mostly on proximal muscles (Horn et al. 2002; Pong et al. 2002). This organization is not maintained in primates, where the RST originates exclusively from mRN and acts on proximal and, preferentially, distal muscles, in particular on the extensor muscles (Belhaj-Saïf et al. 1998).

**Fig. 2:**
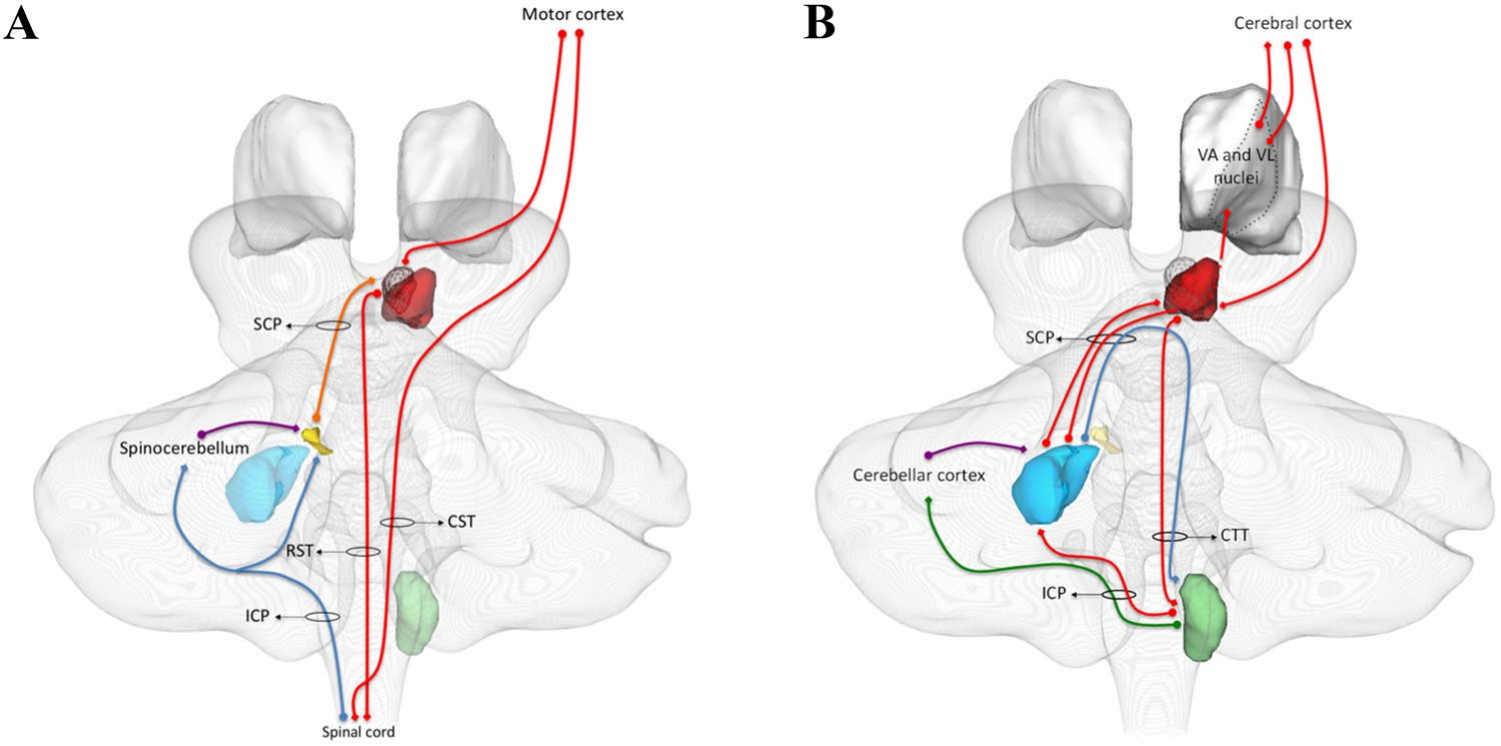
The RN circuitry in detail. 3D rendering of the cerebellum, mRN (edges), pRN (red), dentate nucleus (light blue), interposed nucleus (yellow), inferior olive (green) and thalamus (white). **a** The rubrospinal system. Descending motor cortical output (red arrows) is relayed by the mRN through the rubrospinal tract. Sensory afferents from the spinal (blue arrows) cord reach both the IN and the paravermal cerebellum through the spinocerebellar tracts; cortical cerebellar output converges on the interposed nucleus through Purkinje fibers (purple arrow). Cerebellar output from IN (orange arrow) in turn is relayed on mRN forming a feedback loop. **b** The rubro-olivo-cerebellar system. The pRN receives excitatory afferent fibers (red arrows) from a larger set of cortical regions and from the dentate nucleus, while its main projection output is the rubro-olivary pathway. The inferior olive is connected to the cerebellar cortex via the climbing fibers (green arrow) that synapse directly on Purkinje cells directed to the DN (purple arrow), modulating cerebellar plasticity. Excitatory projections from the inferior olive (red arrows) reach also the dentate nucleus, that in turn sends inhibitory descending projections (blue arrows) forming a feedback loop. *RST* rubro-spinal tract, *CST* cortico-spinal tract, *CTT* central tegmental tract, *ICP* inferior cerebellar peduncle, *SCP* superior cerebellar peduncle, *VA* ventral anterior, *VL* ventral lateral

In addition to the motor function, mRN neurons of both cats and primates respond to sensory stimulation (in particular, light touch, proprioception, pressure and painful pressure), via sensory-encoding neurons that are somatotopically organized into receptive fields (Eccles et al. 1975; Larsen and Yumiya 1980; Kennedy et al. 1986; Matsumoto and Walker 1991). However, sensory responses are weaker than motor responses (Kennedy et al. 1986) and do not influence motor-related discharge (Gibson et al. 1985a). Although a direct spino-rubral pathway has been demonstrated in cats (Padel et al. 1988; Vinay and Padel 1990), it remains unclear whether sensory information comes from direct projections from spinal cord in other animals. Most likely, sensory information is simply relayed from the interposed nucleus, that in turn receives it from the spinocerebellar tracts and shows similarly organized receptive fields (Ekerot et al. 1997). In this regard, it has been postulated that the mRN exerts its role in motor control via a recurrent loop involving the spinocerebellum, interposed nucleus and RST. In this loop, proprioceptive information conveyed to the paravermal cerebellum through the spinocerebellar tracts reach the interposed nucleus and are subsequently relayed to the mRN, thus exerting a feedback control on motor RST efferents (Arshavsky et al. 1988; Houk 1991). Functional anatomy of the rubrospinal system is resumed in Fig. 2a.

Several animal studies outlined the involvement of the rubrospinal system in automatic, complex limb movements. One example is quadrupedal locomotion, that, as seen previously, represented the strongest evolutionary drive for RN differentiation and evolution (ten Donkelaar 1988). Indeed, experiments on decerebrated cats showed that during locomotion, rubrospinal neurons are phasically active preferentially during the swing phase (Orlovsky 1972; Arshavsky et al. 1988), and their microstimulation during walking modifies the activity of physiologically flexor muscles (Rho et al. 1999). More recently it has been hypothesized also a role of the mRN in the maintenance of posture against external perturbations in cats (Zelenin et al. 2010; Herter et al. 2015).

Along with their contribution to locomotion, in rodents, rubrospinal neurons are also involved in controlling skilled movements of the forepaw. This further specialization may have originated from the necessity of avoiding obstacles during walking, as the firing pattern of mRN neurons during this activity is strikingly similar to those of the corticospinal tract (Lavoie and Drew 2002).

Deficits in skilled movements of the forelimb and hand after lesions of the rubrospinal tract are extensively documented on rat models, where the forepaw specializes into hands that can perform rudimentary prehension movements (Whishaw and Gorny 1994; Metz and Whishaw 2000). Excitotoxic lesions of rubrospinal neurons do not impair directly the efficacy of skilled reaching or grasping movements, but selectively interfere with components of the reaching actions, such as limb aiming, hand supination and pronation, and the so-called “arpeggio movement” (gradual opening of fingers after extension of the limb during prehension) (Whishaw et al. 1990, 1992, 1998; Whishaw and Gorny 1994, 1996; Morris et al. 2015). Similar effects are obtained when the RST is transected in the dorsolateral funiculus or at the cervical level (Schrimsher and Reier 1993; Kanagal and Muir 2007, 2008; Morris et al. 2011). It is worth to note that the “arpeggio movement”, a kind of precursor of complex grasping movements of primates (including humans), is selectively impaired by lesions of RST, but not of the corticospinal tract (Whishaw et al. 1998; Kanagal and Muir 2008); this would suggest that, at least in rodents, this movement could be exclusively controlled by the RST.

In primates, the importance of the rubrospinal system in hand movements, such as grasping or manipulation, is evident, as early lesion studies demonstrated deficit in skilled hand movements after mRN lesions (Lawrence and Kuypers 1968). The activity of mRN is extensively correlated with different movements of the upper limbs but discharge rates are higher when movements of the upper extremities are coupled with hand use (Gibson et al. 1985b; Miller et al. 1993; Mewes and Cheney 1994; Belhaj-Saïf et al. 1998; Van Kan and McCurdy 2002a, b). Miller and colleagues showed that a similar number of mRN neurons are active during reaching and grasping, while other limb movements (in this case, the returning of hand to mouth after grasping food) activated a reduced number of neurons. In addition, cross-correlation with electromyographic data revealed that different units within mRN are preferentially related to flexion or extension movements and that their activity is organized in correlation with muscle coordinates (Miller et al. 1993; Miller and Houk 1995).

Other studies highlighted that preferential activity of mRN during reach-to-grasp movements could be related to muscular synergies controlling both flexor and extensor muscles involved in hand pre-shaping (Van Kan and McCurdy 2002a). Further examinations showed that, at least in part, reaching-to-grasp related modulations of discharge rate may contribute to differences in hand pre-shaping connected with target location (Van Kan and McCurdy 2002b).

Taken together these results suggest then a further functional specialization of the rubrospinal system in controlling hand movements. Interestingly, this functional specialization can be observed also for interposed nucleus neurons (Gibson et al. 1994, 1996; van Kan et al. 1994; Geed et al. 2017), highlighting a coherent evolution pattern within the entire functional system. In summary, the primary role of the rubrospinal system in locomotion and postural stability, described in quadrupedal animals, almost disappears in bipedal primates, while the emerging role in controlling hand movements, already present in rodents, further develops towards a more complete specialization.

## Parvocellular RN and the rubro-olivary pathway

In contrast to mRN and the rubrospinal system, that have been extensively characterized in different animal species, functions of the pRN and the rubro-olivary system are less known. This is probably due to the fact that, in other mammals, pRN is less developed and, in any way, not dissociable from its counterpart. On the other hand, in primates, pRN and mRN are anatomically segregated and easily identifiable from electrophysiological recordings, as the former does not show any kind of movement-related activity (Kennedy et al. 1986).

Different animal studies clarified that the inferior olive receives ipsilateral projections from a rich set of mesodiencephalic nuclei (Brown et al. 1977; Saint‐Cyr and Courville 1981; Onodera 1984; Ruigrok and Voogd 1995, 2000), including pRN and other neighboring structures that in rats or cats are considered part of the “extended pRN”, such as the pararubral area (Ruigrok 2004), or the nucleus of Bechterew (Pompeiano and Brodal 1957; Onodera 1984; Horn et al. 2002). The inferior olive sends in turn efferent projections, in form of climbing fibers, that reach the contralateral cerebellar cortex and synapse directly on Purkinje cells with a 1:1 ratio (Eccles et al. 1966; Llinás 2014).

Electrophysiological experiments show that olivo-cerebellar inputs have a characteristic firing pattern (“complex spikes”) (Eccles et al. 1966). Marr (1969) and Albus (1971) proposed that the activation of this system could increase synaptic efficacy of parallel fibers-to-Purkinje synapses, by enhancing synaptic plasticity (Marr 1969; Albus 1971). Purkinje cells project to deep cerebellar nuclei, that in turn project back to the inferior olive, with recurrent projections (Bentivoglio and Kuypers 1982; Teune et al. 1995). In particular, the inferior olive sends excitatory projections to deep cerebellar nuclei, which in turn send inhibitory projections to the IO. Hence, the rubro-olivo-cerebellar system consists of a double feedback/feedforward loop: the IO, that directly modulates Purkinje cells of the cerebellar cortex, has also an excitatory effect on cerebellar nuclei, thus resulting in a feedback inhibition (De Zeeuw and Ruigrok 1994); on the other hand, the mesodiencephalic nuclei of the “extended pRN” send excitatory efferent fibers to inferior olive and receive excitatory afferent fibers from cerebral cortex and deep cerebellar nuclei, establishing a feed-forward loop. (Fig. 2b) (Sotelo et al. 1986; De Zeeuw et al. 1990, 1998; Fredette and Mugnaini 1991; Bazzigaluppi et al. 2012; Llinás 2014). Despite many functions have been attributed to these complex olivo-cerebellar loops, including error sensing, timing and learning of acquired motor behavior, or reflex conditioning, their exact functional roles are highly debated and a clear consensus is still lacking (see De Zeeuw et al. 1998; Lang et al. 2017; Llinás, 2014; Teune et al. 1995 for an exaustive review).

In contrast to the huge amount of studies on the olivocerebellar system, the precise role of pRN and cortico-rubro-olivary projections within this system has not been properly clarified. Early lesional experiments showed that RN lesions disrupt both acquisition and execution of conditioned reflexes (Smith 1970a, b; Rosenfield and Moore 1983, 1985), though evidence is contradictory on whether this structure is preferentially involved in the acquisition or simply in the execution. In this regard, by using a lesion approach, Tsukahara et al. (1981) demonstrated that the corticorubral and rubrospinal pathways alone can be sufficient for the acquisition of a conditioned reflex. The primary neural change for this phenomenon was thought to be a change in the synaptic transmission efficacy at the corticorubral synapses (Murakami et al. 1988), as also demonstrated by further investigations that provided both morphological and electrophysiological evidence of the involvement of corticorubral plasticity in the acquisition of conditioned reflexes (Ito and Oda 1994). By contrast, it has also been suggested that plastic adaptations may take place either in the interposed nucleus or cerebellar cortex, and RN acts simply as a relay involved in the execution of the conditioned response, by controlling rubrospinal or rubrobulbar projections to effector muscles. Arguments in support of this view are that (i) changes of neuronal activity in RN are temporally correlated with the appearance and time course of the conditioned response, suggesting its role in the execution of the response rather than its acquisition (McCormick et al. 1983; Haley et al. 1988; Desmond and Moore 1991); (ii) reversible inactivation of RN seems to have no effect on the response-related activity recorded in the interposed nucleus, whilst inactivation of the interposed nucleus affect the response-related activity of the RN (Chapman et al. 1990); (iii) inactivation of RN exerts its effects both on conditioned and unconditioned eyeblink related responses, while inactivation of the IN prevents the acquisition of conditioned stimuli (Bracha et al. 1993; Krupa et al. 1993). This long-lasting question is still far from being settled, despite recent experiments suggest a more active role for cortico-rubral projections in the acquisition of conditioned reflexes (Pacheco-Calderón et al. 2012).

However, as the vast majority of these experiments have been performed in rabbits and cats, which show peculiar features of rubral neurons (Morcuende et al. 2002; Pong et al. 2002), it is unclear if these results can be translated to primates, where mRN and pRN are more clearly segregated in terms of anatomy and connectivity.

Kennedy and Humphrey (1987) studied the effects of differential lesions at the level of the RN by taking advantage from the property of RST lesions to be almost completely compensated by the corticospinal tract and vice-versa. Two groups of rats were used: the first group was lesioned at the level of the whole RN by using a fiber sparing agent to reduce the interference of fibers of passage. The second group on contrast was first lesioned at the level of the RST, and then received a second lesion in the RN after a few days, after that corticospinal tracts fully compensated the effects of RST transection. While the first group showed absent or partial recovery after lesion, the second group compensated the loss of function almost immediately. The Authors concluded that the rubro-olivary tract may not be necessary in the execution of acquired motor responses, when corticospinal tract has compensated after lesions of RST; vice-versa, impairments on acquired movement execution following lesions of both RST and rubro-olivary tract show no complete functional compensation if they are both lesioned (Kennedy and Humphrey 1987). Results of these experiments, in light of the existing evidence, led Kennedy (1990) to hypothesize that, while pRN and corticospinal tract (in synergy with the olivocerebellar system, corticocerebellar system and dentate nucleus) are active preferentially during motor learning, the mRN and the rubrospinal tract may be involved mainly in the execution of learned or automated movements (Kennedy 1990). This would explain why, after lesions of corticospinal tract in primates, automated movements can be re-acquired by the RST in cooperation with the cortico-rubro-olivo-cerebellar system but, when the olivo-cerebellar tract is lesioned, or both RST and corticospinal tract are severed, the recovery becomes impossible (Lawrence and Kuypers 1968). The rubro-olivary pathway, by exerting an excitatory effect on the olivary nucleus, would then act as a “switch” from automated movements (preferential activity of mRN and the rubrospinal system) to movement learning, mediated by the corticospinal tract and the olivocerebellar system (Kennedy 1990). In this model, it is unclear what kind of input would activate the pRN, regulating the switch from preferential activity of the rubrospinal system to preferential activity of the olivocerebellar system. A more recent experiment conducted in primates suggested that the convergence of cortical and dentate activity on the pRN would encode error information, allowing the activation of the olivocerebellar system during learning from errors (Reid et al. 2009). Although fascinating, however, it should be kept in mind that this model is based on limited experimental evidence and that further research is needed to strengthen these hypotheses.

## Non-motor functions of RN: a role in mediating antinociceptive responses?

Aside to its well-known role in motor behavior, there are some lines of research suggesting that the RN could be also involved in non-motor functions, namely responses to painful stimuli. In rodents, cats and primates, the whole RN contains sensory-encoding neurons that respond to painful stimulation; these responses are, in general, stronger in mRN than in pRN (Nishioka and Nakahama 1973; Eccles et al. 1975; Larsen and Yumiya 1980; Kennedy et al. 1986; Vinay and Padel 1990; Matsumoto and Walker 1991).

In addition, stimulation of the RN in rodents elicits a long-lasting and intense analgesia (Prado et al. 1984), which is probably mediated by sparse anatomical connections of RN with components of the descending antinociceptive system, such as periaqueductal gray, nucleus raphe magnus and lateral reticular nucleus (Gwyn and Flumerfelt 1974; Larsen and Yumiya 1980; Kennedy et al. 1986; Bernays et al. 1988). Probably, this antinociceptive response is regulated on a cellular level by the metabolic pathway of nitric oxide (NO): microinjections of l-arginine (a NO precursor) into RN have a strong antinociceptive effect, that is, in turn, prevented by the inhibition of the NO synthase (Kumar et al. 1995). Since the inhibition of non-NMDA and metabotropic glutamate receptors has also an antinociceptive effect, it is likely that glutamate, the most important neurotransmitter in the RN, plays an opposite effect, by reducing the antinociceptive response (Yu et al. 2015).

However, more recent studies suggest that the molecular regulation of antinociceptive responses in rat RN may be very complex, involving a large network of inflammatory mediators and cytokines. Most of our knowledge about pain regulation in the RN of rodents comes from studies on spared-nerve injury (SNI), an experimental model of persistent peripheral neuropathic pain (Decosterd and Woolf 2000). SNI-induced neuropathic allodynia up-regulates expression of different inflammatory mediators in RN, including Tumor Necrosis Factor-α (TNF-α), interleukin 1-β (IL-1β) 6 (IL-6) and 10 (IL-10), Nerve Growth Factor (NGF) or Transforming Growth Factor-beta (TGF-β) (Li et al. 2008; Wang et al. 2012, 2015, 2016a; Zhang et al. 2013, 2015b; Ding et al. 2018; Guo et al. 2018). Some of these factors, such as TNF-α, IL-1β, IL-6 and NGF, can be inhibited through microinjections of monoclonal antibodies in RN, relieving neuropathic allodynia (Zhang et al. 2013; Wang et al. 2016a; Ding et al. 2018); in addition, these factors can directly induce allodynia in healthy rats when they are injected into RN (Wang et al. 2016a). By contrast, anti-inflammatory mediators such as IL-10 or TGF-β relieve neuropathic pain when injected into RN neurons (Wang et al. 2012, 2015).

Taken together, these results suggest a role for RN in pain induction and modulation. However, neurophysiological and anatomical substrates of this modulatory role are not completely explored at the current state of research. In addition, this role for RN in antinociceptive responses has not been described in other animal species, although limited evidence (that will be discussed further) suggests that RN could exploit similar functions in the human brain.

## The human RN and its unsolved issues

Information about structural and functional organization of RN in human beings is surprisingly lacking when compared with other animals. Historically, this may have been due to many issues that made RN difficult to be studied directly in the human brain. Although these problems have been partially overcome with the rise of non-invasive neuroimaging techniques, the last decades have been characterized by reduced interest in studying human RN anatomy and physiology.

**Fig. 3:**
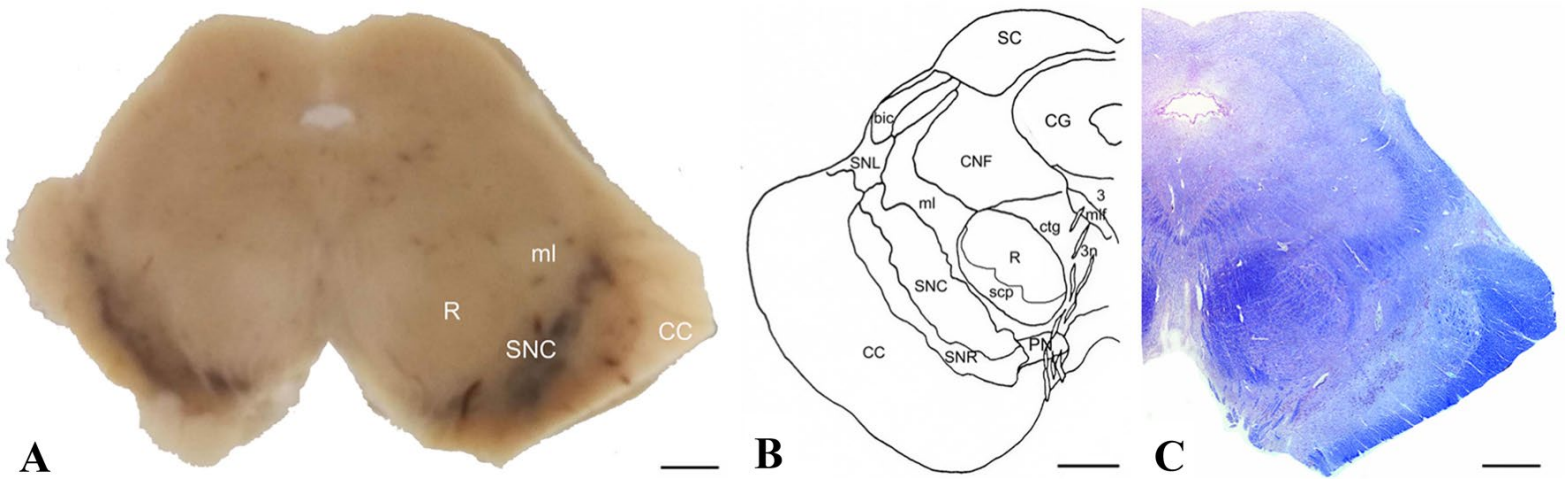
Anatomy of the human RN. **a** Macrophotograph of human autoptic upper midbrain from an adult male of 81 years. **b** Schematic transverse sections of normal human upper midbrain illustrating territorial delineations (adapted from Nieuwenhuys et al. 1980). **c** Right half of a midbrain section from an adult male of 81 years, counterstained with Luxol Fast Blue. *3* nucleus of oculomotor nerve, *3n* oculomotor nerve, *bic* brachium of inferior colliculus, *CC* cerebral crus, *CG* central grey substance, *CNF* cuneiform nucleus, *ctg *central tegmental tract, *ml *medial lemniscus, *mlf *medial longitudinal fasciculus, *PN* paranigral nucleus, *R *red nucleus, *SC *superior colliculus, *scp* superior cerebellar peduncle, *SNC *substantia nigra, compact part, *SNL* substantia nigra, lateral part, *SNR* substantia nigra, reticular part. Scale bar = 2.5 mm

The human RN is located in the ventral midbrain at the level of substantia nigra (SN), it has a roughly spherical shape and is encapsulated by the superior cerebellar peduncle (SCP), which traverses it (Fig. 3). Other white matter tracts passing through RN are the 3rd cranial nerve, that runs along its surface, and the fasciculus retroflexus, that penetrates it at the rostral level delineating a dorso-medial compartment. Many cell bodies are filled with ferric pigment, that probably gives the typical reddish color (Mai and Paxinos 2012). Olszewski and Baxter (1982) subdivided the RN according to its microscopical anatomy into three subregions: a rostral pars oralis, a dorsal pars dorsomedialis and a posterior pars caudalis. Pars oralis is divided from pars dorsomedialis by a thin medullary lamella, while pars caudalis hosts largely interspersed neuronal perikarya (Olszewski and Baxter 1982; Paxinos et al. 2012); these histologically identified regions can be also recognized using high-resolution structural MRI (Abduljalil et al. 2003; Deistung et al. 2013). It is generally believed that the major part of RN is composed by the pRN and that just a few scattered magnocellular neurons are identifiable at the caudal pole of RN (Papez and Stotler 1940; Nathan and Smith 1982; Onodera and Hicks 2009). Recent anatomical studies used incubation with carbocyanine dye, that allowed a clear identification of boundaries of the RN and between pRN and mRN in humans, showing mRN as a small region of large neurons wrapped around the caudal pole of pRN (Onodera and Hicks 2010).

Comparative anatomical studies performed in cat, macaque and man clarified the last steps of the phylogenetical transition to human RN by describing a rolled-sheet model of RN evolution.

In cats, pRN is part of a neural sheet that confines with meso-diencephalic structures such as the nucleus of Darkschewitsch and the nucleus of Bechterew that can be considered as parts of the extended pRN since they project to the inferior olive through the medial tegmental tract (Onodera 1984). This neural sheet in macaque and man “rolls” in a way that the most caudal parts is located rostrally, with the nucleus of Bechterew being located backward to form the dorsomedial part of the pRN. The nucleus of Darkschewitsch is separated from the RN and projects to the inferior olive through the medial tegmental tract, whilst pRN through the central tegmental tract (Onodera and Hicks 2009). By contrast, mRN projects only to the cervical enlargement of the spinal cord through the RST, that crosses the midline and descends in the lateral funiculus, adjacent to the corticospinal tract (Nathan and Smith 1982).

The connectivity of human RN is thought to reflect those of non-human primates. Although rubrospinal, rubro-olivary and cerebello-olivary projections were identified early by classical anatomical dissection (Verhaart 1962), relatively a few studies addressed the topic of anatomical connections of the RN.

In the last decades, technical advances in diffusion tensor imaging and tractography allowed the indirect reconstruction of putative white matter tracts with accurate anatomical detail, non-invasively and in vivo (Cacciola et al. 2016a, b, 2017a, b; Rizzo et al. 2018; Bertino et al. 2020). Tractography has been used to reconstruct known white matter pathways of the human RN, such as the dento-rubral and rubro-olivary tracts (Granziera et al. 2009), but also to explore in detail its structural connectivity profiles. Habas and Cabanis, using diffusion tensor imaging combined with both deterministic and probabilistic fiber tracking algorithms, reconstructed cortico-rubral connections between RN and prefrontal, cingulate, precentral, temporal and occipital cortices. Subcortical connectivity patterns with contralateral and ipsilateral dentate nucleus, and sparsely with putamen and globus pallidus were also identified (Habas and Cabanis 2006, 2007a). Although the existence of direct rubro-pallidal projections, passing through the zona incerta, is in line with early anatomical investigations (Papez and Stotler 1940), a more recent study using advanced diffusion signal modeling algorithms failed in replicating these findings, reporting also cortical connectivity patterns restricted to sensorimotor areas such as superior frontal, precentral, postcentral and paracentral gyri (Milardi et al. 2016). All of these studies, along with intrinsic limitations concerned with low resolution and signal modeling algorithms (Behrens et al. 2007), are not able to differentiate between RN connectivity and passing-by fibers, such as the dento-thalamo-cortical tracts in the SCP. Indeed, passing-by-fibers in the SCP may affect results, as tractography cannot neither distinguish between direct and indirect connectivity nor detect synapses (Jbabdi and Johansen-Berg 2011).

In addition, RST is a very small fiber tract and has a complex decussating course: these features make it hard to be reconstructed with low spatial and angular resolution. Despite these limitations, a preliminary study performed on 21 healthy subjects with 1.5 T MRI and probabilistic tractography reconstructed the RST only in 12 brains bilaterally and 3 unilaterally (Yang et al. 2011). More recently, RST was reconstructed as part of a brainstem white matter atlas using deterministic tractography on a population template obtained from high quality datasets of 466 subjects from the Human Connectome Project (HCP) repository, achieving a complete reconstruction of its origin, decussation and brainstem course (Meola et al. 2016).

Another open question is whether some kind of topographical organization of structural connectivity can be identified within the human RN. A recent study from our research group combined tractography with a connectivity-based parcellation approach to identify topographically organized connectivity clusters within the RN, showing a caudal cluster more connected to interposed nucleus and a rostral cluster more connected to cerebral cortex and inferior olive (Cacciola et al. 2019).

It is generally believed that, in the human brain, mRN and the rubrospinal system are only vestigial residuals without any kind of functional relevance. However, morphological observation in the developing human fetus clearly underline that the development of mRN precedes pRN and that, in the human fetal and neonatal brain, mRN is structurally prominent over pRN (Ulfig and Chan 2001, 2002; Yamaguchi and Goto 2008). These data suggest an intriguing analogy between phylogeny and ontogeny, as mRN and RST could play an important role in the neonatal brain, and its regression in the adult brain can be linked to the transition from quadrupedal to bipedal stance (Hicks and Onodera 2012).

Another interesting clue about the functional role of mRN and the rubrospinal system in the human brain comes from a case series of two PD patients who underwent therapeutic deep brain stimulation (DBS) of the subthalamic nucleus (STN): in such cases, an error of few millimeters in the mediolateral axis lead to the erroneous targeting of RN. Electrophysiological recordings showed that, similarly to STN, RN firing activity resulted to be related to both active and passive movements of the contralateral upper limb and jaws (Rodriguez-Oroz et al. 2008). Despite possible limitations (few subjects studied, pathologic conditions that may have altered RN firing pattern, uncertainty about the precise microelectrode location at the cellular level) this may be the strongest evidence supporting the existence of a functionally active mRN in the adult human brain, as pRN, in primates, does not show any appreciable movement-related activity (Kennedy et al. 1986).

On the other hand, another case report of RN-DBS in a patient with alcoholic cerebellar tremor, reported that RN firing rate was not affected by active or passive movements and showed no tremor-related activity (Lefranc et al. 2014). The Authors suggested that these findings may be due to alcoholic degeneration of deep cerebellar nuclei; another possible explanation could be that authors targeted and recorded pRN activity instead of mRN.

Despite the limited electrophysiological evidence, most of our knowledge about human RN functional activity comes from task-based functional neuroimaging (Habas et al. 2010). An early fMRI study found slight RN activation during passive sensory stimulation and grasping, and significantly higher activity when the task was coupled with an active discrimination task (Liu et al. 2000). Similar results were obtained comparing activations during simple finger opposition and tactile-tactile bimanual discrimination: RN was active both in the pure motor and in the sensory-motor task (Habas and Cabanis 2007b). These studies suggest that, in line with animal research, human RN could play a role both in grasping and finger movements (Van Kan and McCurdy 2002a) and in somatic sensation (Larsen and Yumiya 1980), but add a possible role in active sensory discrimination. In addition, different studies reported bilateral RN activation during both somatic and visceral pain processing (Bingel et al. 2003; Dunckley 2005), thus reinforcing the hypothesis of RN as a nociceptive/antinociceptive structure put forward in rat models (see paragraph above).

Human RN could be also involved in motor control. Early task-related fMRI studies reported contralateral RN activation during execution of both externally triggered and self-initiated sequences of finger movement; in particular, RN activity is stronger during the “planning phase” of self-initiated finger movement in respect to externally triggered movements (Cunnington et al. 2002; Boecker et al. 2008; Habas and Cabanis 2008). A recent approach based on task-related effective connectivity suggested that, during motor preparation, RN functional connectivity is modulated by the presupplementary motor area (preSMA), being part of a cerebello-thalamo-preSMA-RN circuit. Such a circuit would act in synergy with a preSMA-thalamo-caudate nucleus-primary motor area loop during mental concentration before a motor task (Belkhiria et al. 2018). In line with this evidence, another recent task related fMRI study found greater activity bilaterally in RN, STN and SN during failed 'stop' tasks (i.e. subject were receiving an auditory cue to stop them from pressing a button, but pressed it anyway) versus ‘go’ tasks (de Hollander et al. 2017), thus suggesting a strong cooperation between RN and the basal ganglia circuitry in initiation and termination of motor tasks. It is interesting to note that nearly all the mentioned task-related studies were concerned with finger use: this would allow to hypothesize a preferential RN activation when movements of distal upper limbs are involved, in line with its role in animal models.

Another contribution to our understanding of the human RN comes from a few resting-state functional MRI studies. Nioche et al. (2009) showed that RN is functionally connected with a rich set of cortical and subcortical areas, including prefrontal cortex, occipital cortex, posterior hippocampus, caudal insula, thalamus, hypothalamus, basal ganglia and cerebellar lobule V (bilaterally), left precuneus, superior temporal cortex, and dorsolateral prefrontal cortex (only right RN). Another ROI-based analysis described a similar, but less extensive RN functional connectivity network including the dorsal pons, STN, dentate nucleus, SN, insula, dorsal thalamus, putamen, globus pallidus, head of the caudate nucleus, supramarginal gyrus, precuneus and dorsal anterior cingulate cortex (Zhang et al. 2015a). Habas and colleagues used independent-component analysis to disclose cerebellar involvement in resting-state functional networks, and identified at least four functional networks in which RN could be involved: the sensorimotor network, the salience network, the right executive network and the default mode network (Habas et al. 2009).

Taken together, functional neuroimaging studies are in line with animal literature, by suggesting that human RN could be involved in grasping, motor control, somatic tactile and pain perception; however, some works further extend the role of RN in cortico-cerebellar circuitry, suggesting its involvement in higher order functions ranging from sensory discrimination to salience detection, or executive functions. Noteworthy, resting state and task related fMRI studies reporting functional connectivity between RN, cerebellum and basal ganglia structures are in keeping with a growing line of research suggesting a strong interplay between these systems in the human brain (Cacciola et al. 2017c; Caligiore et al. 2017; Milardi et al. 2019; Quartarone et al. 2020). The results of RN neuroimaging research in the last 20 years are summarized in Table 1.

**Table 1:** Twenty years of structural and functional neuroimaging of the RN

Author, year	Subjects	Field strength	Method	Results
Liu et al. (2000)	7	1.9 T	Task-related fMRI	RN activation during passive tactile stimulation; higher RN activation during active discrimination
Cunnington et al. (2002)	12	3 T	Task-related fMRI	RN activation during both self-initiated and externally triggered finger tapping
Abduljalil et al. (2003)	20	8 T	Susceptibility weighted imaging	Macroscopical subdivisions of RN: pars oralis, pars dorsomedialis and pars caudalis
Bingel et al. (2003)	14	3 T	Task-related fMRI	RN activation in response to painful stimuli (laser stimulation)
Dunckley (2005)	10	3 T	Task-related fMRI	RN activation in response to cutaneous and visceral painful stimuli
Habas and Cabanis (2006)	7	1.5 T	DTI, deterministic tractography	RN structural connectivity with prefrontal cortex, premotor cortex, sensorimotor cortex, SCP and CTT
Habas and (2007a)	5	3 T	DTI, probabilistic tractography	RN structural connectivity with prefrontal cortex, premotor cortex, sensorimotor cortex, temporo-occipital cortex, SCP, CTT, globus pallidus
Habas and Cabanis (2007b)	9	3 T	Task-related fMRI	RN activation during bimanual finger-thumb opposition
Boecker et al. (2008)	12	3 T	Task-related fMRI	RN activation during planning and execution of self initiated and externally triggered finger movement sequence; higher RN activation during planning of self-initiated movement
Habas and Cabanis (2008)	7	3 T	Task-related fMRI	Contralateral RN activation during drawing circles (continuous) or triangles (discrete) with pointed index finger
Granziera et al. (2009)	4	3 T	DSI, deterministic tractography	Reconstruction of rubro-olivary and dento-rubral tracts
Habas et al. (2009)	37	3 T	Resting-state fMRI; ICA network analysis	RN involvement in sensorimotor, salience, right executive control and default mode resting state networks
Nioche et al. (2009)	14	3 T	Resting-state fMRI	RN functional connectivity with prefrontal cortex, occipital cortex, hippocampus, claustrum, thalamus, lentiform nucleus, hypothalamus, substantia nigra and cerebellum
Yang et al. (2011)	21	1.5 T	DTI, probabilistic tractography	Reconstruction of rubrospinal tract (only in 32 hemispheres on 42)
Deistung et al. (2013)	9	7 T	Quantitative susceptibility mapping	Macroscopical subdivisions of RN: pars oralis, pars dorsomedialis and pars caudalis
Zhang et al. (2015a, b)	19	3 T	Resting-state fMRI; effective connectivity	RN functional connectivity with STN, DN, SN, insula, dorsal thalamus, putamen, globus pallidus, head of the caudate nucleus, supramarginal gyrus, precuneus and dorsal anterior cingulate cortex; negative modulatory effect of thalamus
Meola et al. (2016)	488	3 T	Q-space imaging; deterministic tractography	Reconstruction of rubrospinal tract and CTT
Milardi et al. (2016)	15	3 T	CSD; probabilistic tractography	RN structural connectivity with cerebellar cortex, thalamus, paracentral lobule, postcentral gyrus, precentral gyrus, superior frontal gyrus and dentate nucleus
De Hollander et al. (2017)	14	7 T	Task-related fMRI	RN activation in stop-and-go trial; increased RN activation in failed stop vs go trials
Belkhiria et al. (2018)	22	3 T	Task-related fMRI; effective connectivity	RN activation in a motor concentration task; effective connectivity cerebello-thalamo-preSMA-RN loop
Cacciola et al. (2019)	100	3 T	CSD; probabilistic tractography; connectivity-based parcellation	RN structural connectivity with cerebral cortex, DN, IN, ION; connectivity based parcellation into two main clusters (Cortex + ION vs IN)

However, care is required in the interpretation of fMRI results about RN, since different limitations may affect the study of midbrain structures. The small size and tightly packed arrangement of midbrain nuclei and the low spatial resolution of fMRI make challenging to obtain a reliable blood oxygen level dependent signal in this area. Moreover, additional noise may come from the cardiac and respiratory cycles (Logothetis 2008). Finally, with these limitations in mind, a registration to a brainstem-optimized template should be carried out in order to properly localize midbrain signals at a group level. In this regard, recently developed, optimized fMRI techniques for brainstem imaging on both 3 T (Limbrick-Oldfield et al. 2012) and 7 T (de Hollander et al. 2017) may help in overcoming these issues and in clarifying the functional role of RN in the human brain.

## From anatomy to clinic: RN in neurological diseases

In the history of neurology, RN has been classically implied in the pathophysiology of tremor, since Gordon Holmes, in 1904, hypothesized that lesions of the RN could lead to a characteristic tremor type, the so-called “Holmes tremor” (HT) or rubral tremor (Holmes 1904). However, several case reports suggest that HT may arise from different lesions, not necessarily involving the RN (Rieder et al. 2003; Raina et al. 2007, 2015, 2016). Currently, the most commonly accepted pathophysiological hypothesis postulates a “double-hit” pathogenesis of HT, that may be due to contemporary lesion of dopaminergic nigro-striatal projections and cerebellar dento-thalamic fibers (Remy et al. 1995; Shepherd et al. 1997; Rieder et al. 2003; Gajos et al. 2010). More recent reports described that infarction of RN may manifest with both motor cerebellar symptoms (tremor, asynergia, adiadochokinesia, dysmetria) and non-motor symptoms (memory impairment, decreased verbal fluency, intellectual fatigability) (Lefebvre et al. 1993). However, as RN lesions often involve also neighboring structures, these clinico-pathologic correlations should be interpreted with care (Pérez-Balsa et al. 1998).

Another form of tremor that has been historically related to lesions in the dento-rubro-olivo-cerebellar pathway is the oculopalatal tremor (OPT) (Guillain and Mollaret 1931). This kind of tremor may arise from lesions involving the dento-rubro-olivary pathway, in particular at the level of dentate nucleus or central tegmental tract, and is frequently associated with hypertrophic olivary degeneration (HOD) (Tilikete and Desestret 2017). However, animal models of OPT/HOD suggest that the RN may be not directly involved in OPT, that would instead derive from lesion of inhibitory projections from deep cerebellar nuclei to inferior olive via the central tegmental tract. Inferior olive denervation would cause hypertrophy and disinhibition, altering the normal tonic firing pattern, thus likely resulting in abnormal olivo-cerebellar feedback manifesting as tremor (Sotelo et al. 1974; Ruigrok et al. 1990; Shaikh et al. 2010; Tilikete and Desestret 2017).

RN and its connections have also been long-time regarded as involved in the pathophysiology of essential tremor (ET). Early PET findings demonstrated metabolic hyperactivity in RN, along with thalamus, cerebellum, DN and striatum, in patients with ET (Wills et al. 1994, 1995). In addition, alteration of diffusion parameters of RN, SCP and dentate nucleus have been interpreted as early pathological changes that may underline tremor symptoms (Jia et al. 2011; Shin et al. 2008).

Although RN appears to be strictly tied to both olivocerebellar and cerebello-thalamo-cortical systems, which have been implied in the pathophysiology of ET (Simantov et al. 1976; Llinás and Yarom 1986; Sharifi et al. 2014; Louis and Lenka 2017) its precise role on the onset and development of ET symptoms is yet to be clarified.

Moreover, recent findings suggest that RN might be involved in PD. Human RN is rich in iron, and oxidative stress subsequent to alteration in iron storage and metabolism has been considered as a potential mechanism for neuronal cell death and pathological features in PD (Hirsch and Faucheux 1998; Rhodes and Ritz 2008).

Several studies using advanced MRI techniques for iron detection and quantification reported progressive accumulation of iron in different brain nuclei, including RN. However, this finding has never been confirmed neither discarded by neuropathological analysis and it remains unclear if iron content in RN increases or decreases during PD course (Martin et al. 2008; Barbosa et al. 2015; Wang et al. 2016b; Guan et al. 2017). It has been suggested that, while in the early stages of disease only substantia nigra pars compacta is affected, substantia nigra pars reticulata, globus pallidus and RN are affected in advanced PD stages (Guan et al. 2017). The pathophysiological meaning of this phenomenon is still unclear: as iron content of RN was correlated with development of levodopa-induced dyskinesia, it has been hypothesized that it may underlie a cerebellar motor compensation mechanism after treatment with levodopa (Wang et al. 2016b; Guan et al. 2017). Finally, Lewis et al. (2013) demonstrated a correlation between increased iron in dentate nucleus and RN with tremor symptoms in advanced PD patients.

It can be concluded that, although evidence ties RN with tremor and tremorgenic syndromes, the RN role in tremor generation is still to be better elucidated. Nevertheless, it has been suggested that RN could represent a suitable DBS target to treat tremor, as the outcome from a single case of RN-DBS for cerebellar alcoholic tremor gave encouraging results on the postural component of tremor (Lefranc et al. 2014).

Notably, also the mRN and rubrospinal tract may have clinical implications in humans.

Traditionally, the involvement of RN, in particular of its magnocellular portion and of the rubrospinal tract, has been implied in mediating the clinical differences between decorticate and decerebrate rigidity (Ward 1947; Carey et al. 1971), as commonly stated also in modern-days neurology and neurosurgery textbooks (Whitney and Alastra 2020).

In experimental animal models, decerebrate rigidity, characterized by extension of the lower and upper limbs, generally develops due to transection of the brainstem from the level of superior colliculus to the level of vestibular nuclei. By contrast, lesions above the superior colliculus may manifest with decorticate rigidity, that involves a flexor response of the upper limbs (Sherrington 1898; Bazett and Penfield 1922; Ranson and Hinsey 1929a).

Earlier experiments suggested that a lesion damaging the RST, which has a facilitatory effect on flexor muscles, may cause the extension of the upper limbs in decerebrate rigidity, while damage in cortico-rubral tracts, and the following disinhibition of the RN, are likely to account for the flexor response in decorticate rigidity (Rademaker 1931). However, this view has been challenged by different early experiments which failed to replicate flexor or extensor rigidity after ablation, stimulation or lesion of the RN (Ranson and Hinsey 1929b; Ingram and Ranson 1932; Ingram et al. 1932; Keller 1934); moreover, additional experimental studies on the decerebrate animal suggest that extensor rigidity may rather emerge from lesions in the pontine and bulbar reticular formation (Ward 1947).

In humans, there is general agreement that decerebrate posturing may emerge from lesions of the brainstem, while decorticate posture often follows lesions at the level of the cerebral cortex, basal ganglia or thalamus. However, frequent clinical, pathological and radiological overlaps between these two syndromes are reported (Turazzi and Bricolo 1977; Davis and Davis 1982; Klug et al. 1984; Woischneck et al. 2015). These clinical and radiological features make very difficult to attribute the presence or absence of symptoms (such as flexor or extensor rigidity) to the involvement of specific brain regions.

**Fig. 4:**
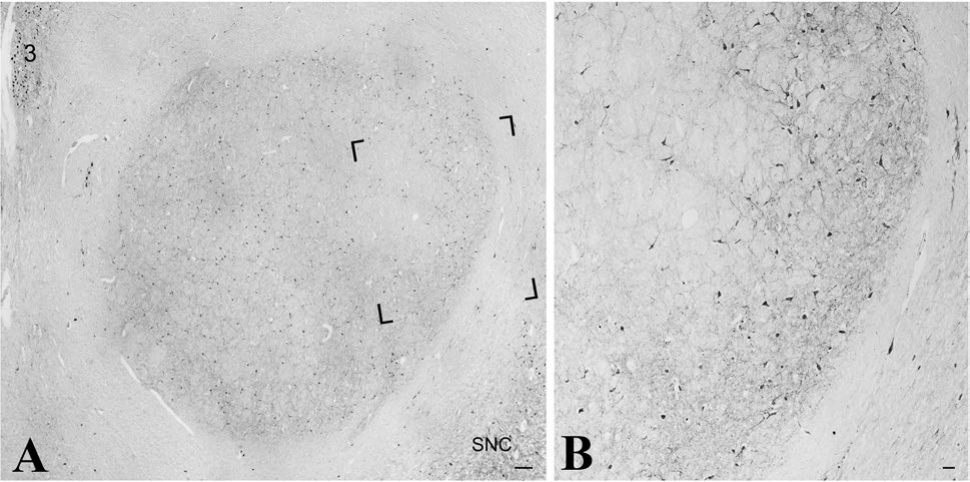
BDNF expression in adult human RN. Human adult midbrain from an adult female of 67 years immunostained for BDNF. **a** Low-power view photomontage of the right red nucleus. **b** Higher magnification of the area framed in (**a**). *3* nucleus of oculomotor nerve, *SNC* substantia nigra, compact part. Scale bars: **a** = 250 μm; **b** **=** 50 μm

In addition to the controversial role of mRN and rubrospinal tract in decerebrate and decorticate rigidity, early primate studies have highlighted their possible role in replacing the corticospinal tract after pyramidal lesions (Lawrence and Kuypers 1968). In primates, when the corticospinal tract is damaged, rubrospinal neurons may undergo plastic modifications to rearrange their facilitating effects on both flexors and extensor muscles (Belhaj-Saïf and Cheney 2000). Similarly, in rodents the RST is able to compensate the loss of motor function following pyramidal tract transection at the spinal level (Kennedy and Humphrey 1987); putative mechanisms of neuronal plasticity, including axonal sprouting and collateralization, and the possible role of Brain-Derived Neurotrophic Factor (BDNF) in mediating these processes, have been extensively studied in murine models of spinal cord lesions (see Morris and Whishaw (2016) for review). However, these studies are not entirely generalizable to the human brain, where mRN is considerably smaller and RST is clearly less developed than in other animal species (Nathan and Smith 1982; Patt et al. 1994). On the other hand, it is relevant to note that human adult rubral neurons have been reported to be immunoreactive to BDNF (Fig. 4) (Quartu et al. 2010), thus indicating that this neurotrophin may represent the molecular substrate for neuroplastic adaptive responses. In addition, it should be noted that electrophysiological evidence suggests that mRN can be functionally active in the human brain (Rodriguez-Oroz et al. 2008) and that the recovery of motor functions, rather than by mRN and RST alone, may be synergistically mediated by both the rubrospinal and rubro-olivary system (Kennedy 1990).

In chronic post-stroke patients, structural connectivity between primary and supplementary motor cortices and RN is significantly correlated with behavioural measures of upper extremity functions, suggesting a reorganization of the cortico-rubral system in the recovery of upper limb motility (Rüber et al. 2012). Increased fractional anisotropy in RN, rubrospinal and cortico-RN tracts was found to be positively correlated with the level of motor impairment in chronic post-stroke patients at different time intervals after lesion, likely suggesting structural reorganization and plasticity (Yeo and Jang 2010; Rüber et al. 2012; Takenobu et al. 2014; Jang and Kwon 2015; Kim et al. 2018). This hypothesis is strengthened by fMRI findings of increased activation of cerebral cortex, cerebellum and RN that correlated with recovery of motor functions after a treadmill gait exercise (Luft et al. 2008). However, further investigations are required to confirm RN potential in neurorehabilitation.

Finally, to complement our overview on the possible pathophysiological implications of the human RN, it is worth to mention that some lines of evidence suggest the involvement of RN in migraine, in keeping with its possible role in the nociceptive/antinociceptive system. Recent pathophysiological theories suggest that migraine may be a disorder of the neurovegetative and nociceptive brainstem, and that local imbalances in the activity of hypothalamic and brainstem circuitry could be implied in the initiation and termination of migraine attacks (May 2017). Although the involvement of RN in such circuitry in the human brain is yet to be clarified, some fMRI studies report intense activation and hyperoxia of bilateral RN during both spontaneous and visually-triggered migraine (Welch et al. 1998; Cao et al. 2002; Aurora et al. 2004). More recently, resting-state fMRI has been used to study functional connectivity of brainstem structures in migraineurs, reporting altered functional connectivity between RN, parietal lobe and cerebellum (Huang et al. 2019). Finally, iron accumulation in RN has been described in different cohorts of chronic migraineurs (Kruit et al. 2009, 2010; Domínguez et al. 2019), despite not consistently replicated by other studies (Tedeschi et al. 2013; Palm-Meinders et al. 2017; Skorobogatykh et al. 2019). Hence, a better understanding of RN involvement in nociceptive circuits could lead to a better comprehension of its role in migraine and other pain-related syndromes.

## Conclusion: what we know and what we have to learn

The present work aimed at providing a comprehensive overview on RN structure and function, connecting the dots from animal and human studies, in order to bridge the gap between basic and translational research. The path towards a better understanding of RN role in the human brain is not free from inconsistencies and misunderstandings, and many steps still divide us from a complete comprehension. However, we believe that some sparse firm points can be inferred from our review:

- phylogenetically the RN is likely to be correlated with the appearance of limbs or limb-like structures, and it plays an important role in the transition from aquatic to terrestrial locomotion;
- the primitive RN functionally corresponds to mRN and is involved mostly in locomotor functions and in the execution of simple, stereotyped movements; the pRN develops later and is probably related to increasing behavioral complexity;
- although already present in quadrupedal animals, mRN functional specialization for upper limb movements becomes more evident with the evolution of bipedalism;
- it is still unclear if mRN undergoes a complete regression in the adult human brain, but it is possible that it can play a role during human ontogenesis and in the recovery of pyramidal lesions;
- functions of pRN are still largely unknown in animal models, but it can be hypothesized that they may control the motor system on a hierarchically higher level, in cooperation with the olivo-cerebellar and basal ganglia systems;
- a few findings from rat models and human neuroimaging imply a role for RN in mediating antinociceptive responses to pain stimulation.

The main uncertainty about human RN is that animal findings cannot be fully translated into human research.

In our opinion, determining whether the human RN is organized (as in other animal species) in two different structurally and functionally dissociable compartments is a fundamental issue that needs to be addressed. It is clear from animal models that mRN and pRN, despite being probably part of a strictly interconnected system, play different functional roles. While most of traditional basic research suggests that only pRN has a functionally relevant role in the human brain, clinical and applied neurosciences argue against this assumption, suggesting that also the phylogenetically older portion of RN may still have a relevant part in human physiology and pathophysiology. Hence, as no conclusive evidence is provided, we believe that further studies in this direction could offer the answers needed to settle the debate.

Neuroimaging remains the elective tool for studying, in vivo and non-invasively, the human RN in both physiological and pathological conditions, and the advances made in the last decade could overcome the intrinsic technical limitations that are typical of the earlier studies.

A detailed multi-modal MRI atlas of the human RN appears to be the only tool that could help bringing together results from histology, high-field MRI, functional and structural connectivity. Improvements in neuroimaging of the RN could also facilitate our understanding of its role in different brain systems, and to clarify how it cooperates with other brain functional structures, such as cerebellar and cerebral cortex, or the basal ganglia, in the execution of complex tasks.

Clinicians may take advantage from basic research to understand how RN dysfunction can lead to various symptoms. Hence, innovations in the field of basic research may pave the way for more detailed insights on the role played by RN in the pathophysiology of different brain diseases and may help in bridging the gap between basic and translational research.

Interest about the RN has decreased in the last twenty years. In the present review, we demonstrate that the neuroscience field has still something interesting to tell about this neglected structure. We believe that our review may facilitate further research by providing a comprehensive perspective on human RN, from its phylogenesis and development to its pathophysiological implications, and by highlighting lacking or faulty knowledge. We hope this may boost a renewed interest towards the RN and act as a trigger for further research, in order to better understand its functional role in the human brain.

## Data Availability

Online sources including PubMed/MEDLINE, Google Scholar, EMBASE have been used for the purpose of the review.
